# How to Deal with Skin Biopsy in an Infant with Blisters?

**DOI:** 10.3390/dermatopathology8020022

**Published:** 2021-06-04

**Authors:** Stéphanie Leclerc-Mercier

**Affiliations:** Reference Center for Genodermatoses (MAGEC Center), Department of Pathology, Necker-Enfants Malades Hospital, Paris Centre University, 75015 Paris, France; stephanie.leclerc@aphp.fr

**Keywords:** blistering eruption, infant, skin biopsy, genodermatosis, SSSS, hereditary epidermolysis bullosa, keratinopathic ichthyosis, incontinentia pigmenti, mastocytosis, auto-immune blistering diseases

## Abstract

The onset of blisters in a neonate or an infant is often a source of great concern for both parents and physicians. A blistering rash can reveal a wide range of diseases with various backgrounds (infectious, genetic, autoimmune, drug-related, traumatic, etc.), so the challenge for the dermatologist and the pediatrician is to quickly determine the etiology, between benign causes and life-threatening disorders, for a better management of the patient. Clinical presentation can provide orientation for the diagnosis, but skin biopsy is often necessary in determining the cause of blister formations. In this article, we will provide information on the skin biopsy technique and discuss the clinical orientation in the case of a neonate or infant with a blistering eruption, with a focus on the histology for each etiology.

## 1. Introduction

The onset of blisters in a neonate or an infant (<2 years old) is a source of great concern for both parents and physicians. Therefore, a precise diagnosis, between benign causes and life-threatening disorders, is quickly needed for the best management of the baby.

Several diseases with various backgrounds (infectious, genetic, autoimmune, drug-related, traumatic, etc.) can lead to a blistering eruption.

Skin biopsy is often necessary in determining the cause of the blister formation.

In the present article, only the most frequent etiologies specific to this age group will be described, with a special emphasis on histology in the clinical context (Table 1).

Vesicular and pustular eruptions will not be discussed in this review.

### 1.1. How Can the Clinical Context Provide Information Concerning the Etiology?

The underlying context of blistering lesions in newborns or infants will sometimes be helpful to provide a clinical orientation. Some elements important to investigate are listed below with examples of etiology:

- distribution of the lesions: localized blisters (traumatic/suction blister, local infection, solitary mastocytosis, etc.)
- general symptoms: fever, systemic disorders (infection, toxic epidermal necrosis, metabolic disorder, etc.)
- tense or flaccid blister/erosion (giving indications on the level of splitting)

### 1.2. What Is the Appropriate Technique for Skin Biopsy in This Context?

Even if invasive, a skin biopsy is often necessary in this situation for a quick diagnosis and a better management of the patient. 

The physician should give the pathologist a precise report with the clinical data, the precise topography of the biopsy, the anesthetic procedure, and a description of the lesion collected. The injection of an anesthetic product will be performed, but an anesthetic cream containing lidocaine prilocaine should not be applied prior to the biopsy since it can be responsible for histopathologic changes and lead to misdiagnosis, and also prevent ultrastructural analysis (vacuolization of keratinocytes) [1].

A skin biopsy will be taken for routine examination, and another will be frozen for immunohistochemistry techniques (split skin is not usually performed in this acute situation). A biopsy for electron microscopy is usually not routinely available but could be useful for genodermatosis diagnosis. 

**Figure 1:**
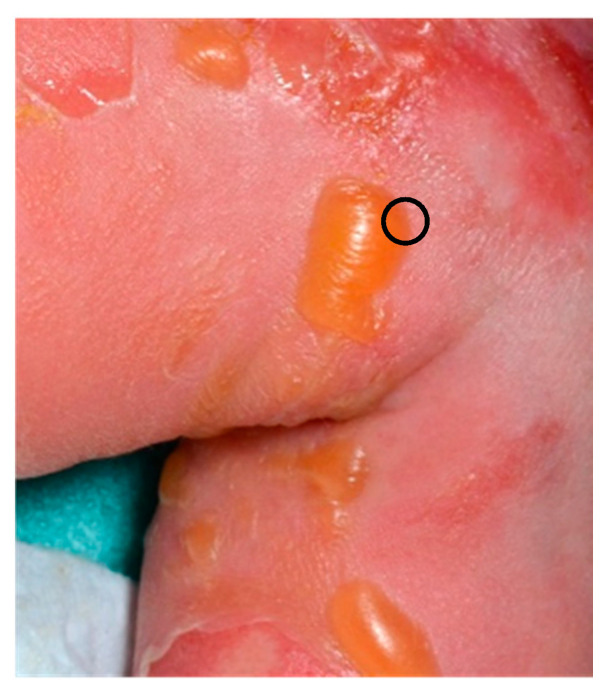
Skin biopsy for EBH, between normal skin and the edge of the blister.

The biopsy should be taken on the most recent lesion within 24 h (and within 12 h when possible):

- for routine histology, it must be performed between the normal skin and the edge of the blister so that the epidermis does not detach as in Figure 1, fixed in formalin, and processed routinely (paraffin embedded, haematoxylin and eosin (HE) staining) in order to determine the anatomic level of the blister
- for immunohistochemistry, it will be frozen or can be put in Michel media
  ○ if an autoimmune blistering disease (AIBD) is suspected, the biopsy will be performed on healthy perilesional skin and processed for direct immunofluorescence (DIF)
  ○ if a genodermatosis is suspected, the biopsy will also be taken between the normal skin and the edge of the blister and processed for immunomapping with specialized antibodies (DIF or immunoperoxidase)

## 2. Etiologies

### 2.1. Infections

Infections are the first cause of blistering eruption in neonates and infants.

#### 2.1.1. Staphylococcal Scalded Skin Syndrome (SSSS)

SSSS is an exfoliative, toxin-mediated eruption caused by certain strains of *Staphylococcus aureus* producing an epidermolytic toxin (formerly named exfoliatin). The disease affects mostly neonates and children under 5 years of age but can also affect adults. In neonates, omphalitis is often the origin of the infection [2]. 

**Figure 2:**
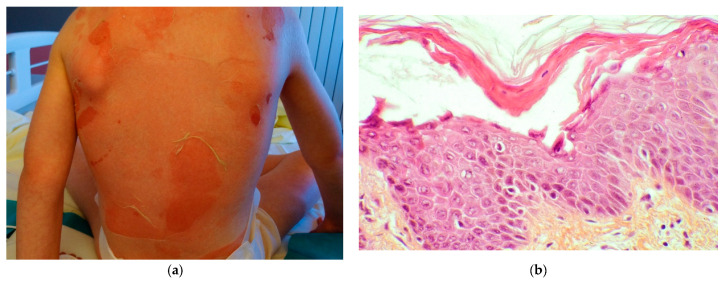
SSSS. (**a**) Clinical appearance of SSSS with Nikolsky sign. (**b**) Hematein eosin ×40: subcorneal blister with acantholysis.

SSSS usually presents with prodromes (irritability, malaise, and fever) followed by a painful erythema, blistering with large areas of epidermal detachment, beginning on the face, neck, axillae, and groin. SSSS is associated with a positive Nikolsky sign (Figure 2a). Mucosae are not affected. 

The toxin in SSSS (and also bullous impetigo) causes a cleavage in the granular layer.

Skin biopsy is not always performed except in case of toxic epidermal necrosis (TEN) suspicion.

#### 2.1.2. Histology

A subcorneal blister is observed along the granular layer and accompanied by a mild inflammatory infiltrate in the epidermis or dermis. Acantholysis is often present, as shown in Figure 2b, and except in the situation of an old blister, no necrosis is observed. Dermal edema and blood vessel dilatation can be observed in the superficial dermis. 

In TEN, the blister is subepidermal and epidermal necrosis is present; a frozen section can be performed for quick results [3]. 

#### 2.1.3. Impetigo

**Figure 3:**
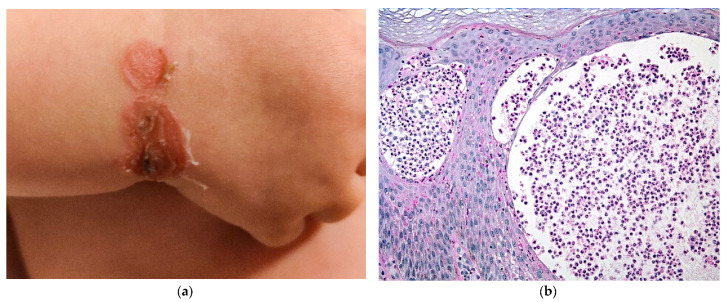
Impetigo. (**a**) Post-bullous lesion of impetigo. (**b**) PAS staining × 20: Impetigo, vesicles filled with neutrophils.

Bullous impetigo usually affects children between 2 and 5 years of age but can be observed in infants. The disease is usually transmitted through direct contact and cutaneous trauma is often found. Bullous impetigo is considered as a localized form of SSSS and caused by the same strains of *S. aureus* producing an epidermolytic toxin. Superficial vesicles progress to a flaccid blister replaced with yellow crusting after rupture with a peripheral peeling, which is helpful for the diagnosis (Figure 3a). Bullous impetigo is often located in infants in moist areas, such as the perineum, axillae, and neck folds, sparing the mucosae [4]. 

#### 2.1.4. Histology

Skin biopsy is rarely necessary. As in SSSS, the cleavage is subcorneal and the blister usually contains neutrophils (Figure 3b), acantholytic cells, and sometimes Gram-positive germs. 

Superficial dermis is inflammatory (lymphocytes and neutrophils). 

The main differential diagnosis of impetigo is superficial pemphigus in cases with acantholytic cells [5].

#### 2.1.5. Scabies

**Figure 4:**
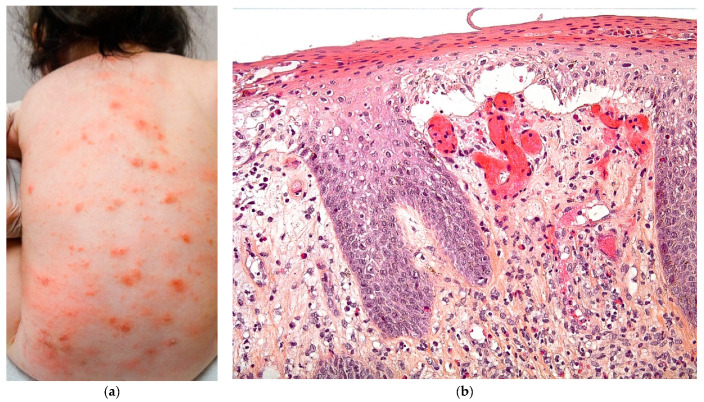
Scabies. (**a**) Blistering scabies. (**b**) Hematein eosin ×20: dermo-epidermal blister and inflammatory infiltrate with eosinophils in the dermis.

Scabies is a contagious disorder caused by the infestation of the skin with *Sarcoptes scabiei* var. *hominis*. It can occasionally have a blistering presentation, as in Figure 4a.

In infants, palms and soles are often involved as well as the sides of the feet.

The presence of an individual or familial pruritus, often during night, provides clinical orientation [5]. 

#### 2.1.6. Histology

A skin biopsy is not performed to confirm the diagnosis of scabies but usually because of the suspicion of another diagnosis, such as an autoimmune blistering disease.

The epidermis is acanthotic with an important spongiosis resulting in vesiculation and blistering (Figure 4b), with exocytosis of eosinophils and neutrophils. Serial cuts can reveal the female mite and her eggs in the stratum corneum. An important inflammatory cell infiltrate is present in the dermis, along the vessels and adnexae, made of lymphocytes, histiocytes, and eosinophils. Langerhans cells may be numerous and sometimes, if an immunohistochemistry with anti-CD1a antibody is performedit can be misleading and result in a Langerhans cell histiocytosis diagnosis. Flame figures are often present in infants. 

### 2.2. Genodermatosis

#### 2.2.1. Hereditary Epidermolysis Bullosa

Inherited epidermolysis bullosa (EB) comprises a highly heterogeneous group of rare diseases characterized by fragility and/or blistering of the skin and mucosae. EB type and subtype classification is presently defined by “onion skin”, terminology based on the combination of level of skin cleavage corresponding to the major EB type, the clinical severity, the inheritance pattern, and the molecular defect, including the relative protein expression and the disease-causing sequence variant (Table 2) [6]. 

There are four main subtypes: EB simplex (EBS), junctional EB (JEB), dystrophic EB (DEB), and Kindler syndrome [7]. 

In these four subtypes, mucocutaneous fragility is the result of abnormalities of the cutaneous basement membrane zone (BMZ) (hemidesmosomes, focal adhesions, anchoring filaments, and anchoring fibrils) [8]. 

**Figure 5:**
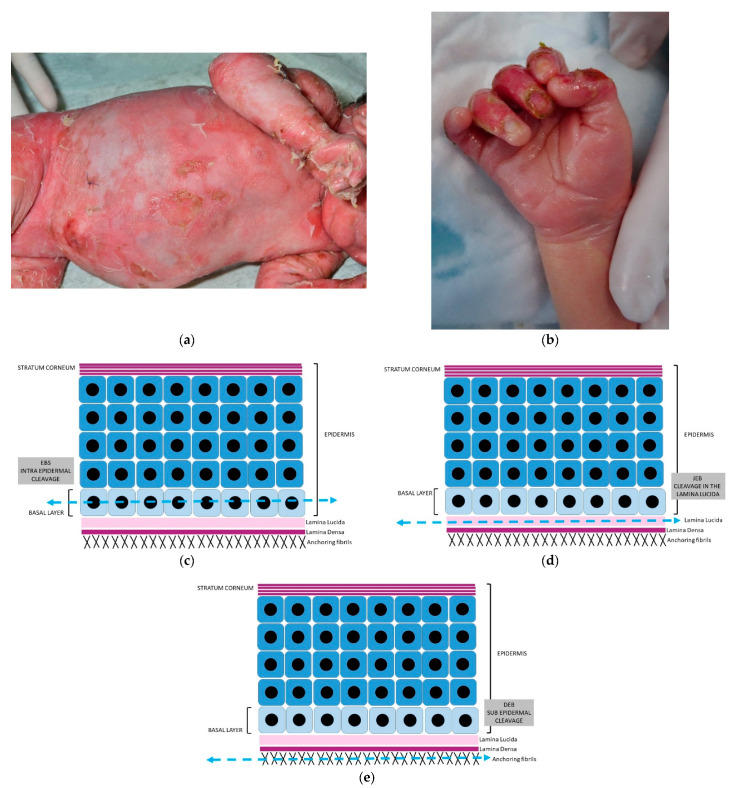

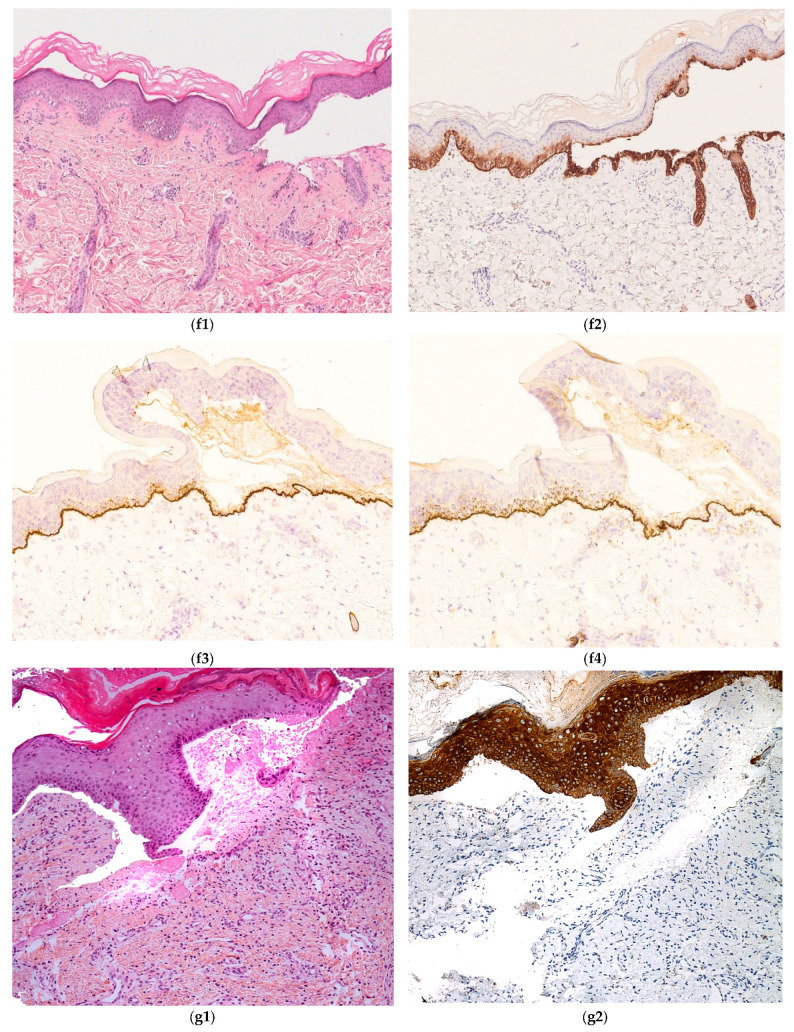

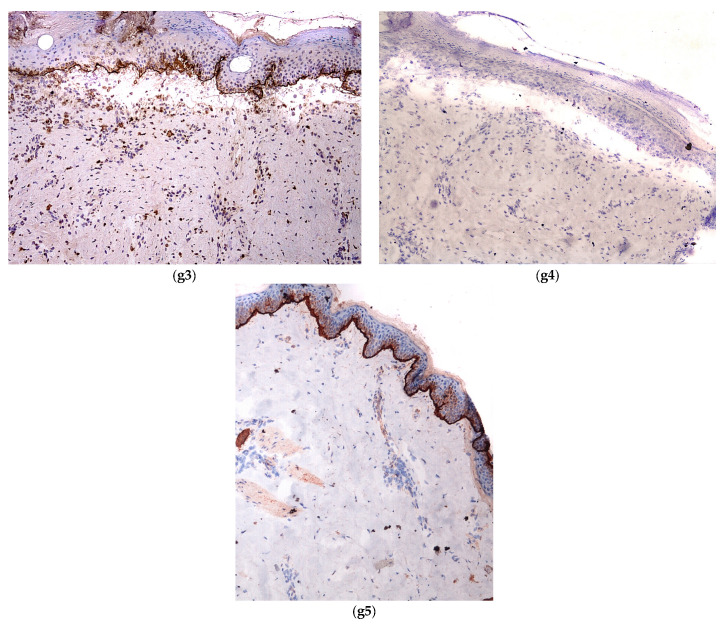
Hereditary epidermolysis bullosa. (**a**) Generalised blisters in EBS. (**b**) Cutaneous aplasia in EBJ. (**c**) EBS: intraepidermal cleavage. (**d**) EBJ: cleavage in lamina lucida. (**e**) EBD: cleavage in anchoring fibrils. EB simplex: (**f1**) Hematein eosin ×10: blister at the DEJ with vacuolization of basal keratinocytes (arrows) and remnants of basal keratinocytes (asterisk) on the floor of the blister; (**f2**) immunohistochemistry with anti-pan cytokeratin antibody showing positivity on the roof and the floor of the blister; (**f3**,**f4**) immunohistochemistry with anti-laminin and anti-collagen VII antibodies showing positivity on the floor of the blister. Dystrophic EB: (**g1**) Hematein eosin X10 blister at the DEJ; (**g2**) immunohistochemistry with anti-pan cytokeratin antibody showing positivity on the roof of the blister; (**g3**) immunohistochemistry with anti-laminin showing positivity on the roof of the blister; and (**g4**) anti-collagen VII antibodies showing absence of expression compared to a control (**g5**).

The clinical appearance depends on the level of skin cleavage: superficial blisters or erosions will occur with EBS, and blisters will be more profound with JEB, DEB, and KS, and lead to ulcerations. Blisters may be generalized or localized to the extremities, as shown in Figure 5a,b.

The skin defects heal with dyschromia, atrophy, and scarring. Mucosae and skin adnexa (nails, hair) are also affected. 

A correct and rapid diagnosis of EB type is mandatory for an optimal management of the baby, for parent information about prognosis, for therapeutic options, and also for genetic counseling.

Immunomapping is the first diagnostic step as it can deliver rapid results in less than 48 h [8]. 

#### 2.2.2. Histology

The biopsy (see technique above) must be taken in a fresh lesion because, after a short delay, re-epithelialization occurs, and the real level of the blister will be difficult to assess.

Standard histology will usually show a subepidermal blister in all subtypes; the epidermis is usually intact, except in an old biopsy in which necrosis of the epidermis can be observed. In EBS, eosinophilic granules corresponding to tonofilaments clumping may be present, as well as modifications of basal keratinocytes (vacuolisation) and occasionally keratinocyte remnants are present on the floor (Figure 5(f1)).

The blister is usually empty without inflammatory cells when recent, but inflammation can occasionally be observed. The floor of the blister is made of dermis with conservation of the papillary relief and no or little inflammation, except in the case of generalized EBS.

**Figure 6:**
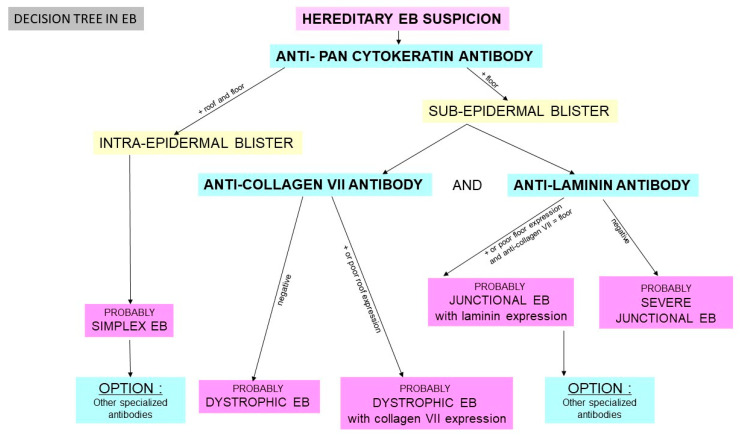
Decision tree.

Immunomapping will be performed on the fixed and frozen biopsies to assess the level of cleavage of the blister (Figure 6).

The location of the cleavage is detailed in Figure 5c–e.

In general pathology laboratories, immunohistochemistry with anti-pan cytokeratin and anti-collagen 4 antibodies can be used to provide rapid results.

In specialized centers, three main antibodies can be introduced as a first step for immunomapping because they are necessary for diagnosis of the three main EB subtypes (EBS, JEB, DEB):

- anti-pan cytokeratin AE1/AE3
- anti-laminin antibody
- anti-collagen VII antibody

If necessary, other specialized antibodies are introduced to study the other proteins of the DEJ (anti-integrin, anti-plectin, anti-BP180).

Figure 5(f1–g5) show examples of immunomapping in two different subtypes of EB.

#### 2.2.3. Keratinopathic Ichthyosis (KI)

KI is a new umbrella term defined by the First Ichthyosis Consensus Conference in Sorèze in 2009, which revised the nomenclature and classification of inherited ichthyoses [9]. 

KI encompasses ichthyoses caused by keratin mutations, namely epidermolytic ichthyosis (EI), superficial epidermolytic ichthyosis (SEI), and other non-blistering variants. 

**Figure 7:**
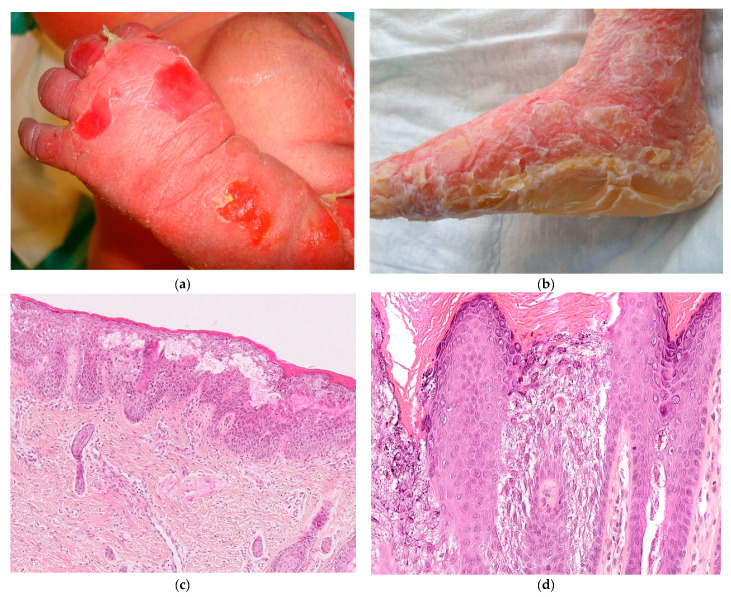

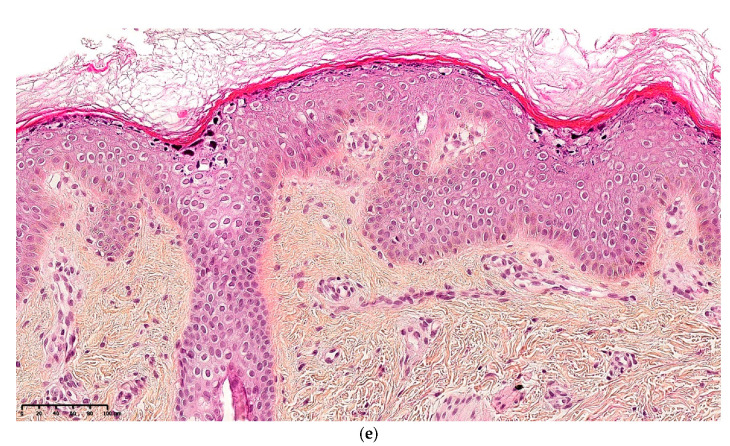
Epidermolytic ichthyosis. (**a**) Newborn with superficial blisters and erythroderma. (**b**) Palmoplantar keratoderma in *CK1 mutation.* (**c**) Hematein eosin ×10: confluent epidermolysis leading to blister. (**d**) Hematein eosin ×20: epidermolysis with basophilic and eosinophilic granules. (**e**) Hematein eosin ×20: SEI, epidermolysis is located in the granular layer.

Newborns may show widespread blistering resembling epidermolysis bullosa (Figure 7a), or have severe erythroderma requiring a specific management, so an early diagnosis is therefore necessary. 

A correct diagnosis of ichthyosis is essential for genetic counseling but also for patient information about prognosis and therapeutic options. 

These rare keratinization disorders are due to pathogenic variants in the genes encoding keratins 1, 2, or 10. Mutations result in keratin clumping and collapse of the cytoskeleton in the suprabasal keratinocytes (keratin 1 or 10) or upper layers (keratin 2).

The presence of palmoplantar keratoderma (Figure 7b) provides clinical orientation for keratin 1 mutations as keratin 10 is absent in palms and soles.

#### 2.2.4. Histology

The histological diagnosis of EI will easily and quickly help the physician, since the appearance of epidermolysis is visible at a low magnification with the typical appearance of epidermolytic hyperkeratosis [10,11,12], with vacuolated suprabasal keratinocytes, affecting the upper part or the entire epidermis with or without a blister formation (Figure 7c). The stratum corneum can become very thick following the first days of life, sometimes compact or more basket-weave like. The granular layer is also affected, containing basophilic granules and the keratinocytes containing eosinophilic granules, corresponding to clumps of tonofilaments in electron microscopy (Figure 7d).

Interestingly, the diagnosis of KI can be performed very early in life and the diagnosis can be very fast in a newborn just by the examination of a frozen section, therefore eliminating the other differential diagnosis of extensive skin blisters, such as infection or hereditary epidermolysis bullosa. In our experience, the clinical appearance does not correlate with the intensity of histologic abnormalities.

Recently, Galler et al. [13] reported the opportunity of a quick diagnosis with minimal trauma to the patient by performing the “jelly-roll” technique. The observation of the roof of a blister shows characteristic findings of epidermolytic hyperkeratosis, which included hyperkeratosis with granular layer degeneration, vacuolization, and eosinophilic globules. The authors suggest that this technique can be used for the diagnosis of EI. 

The histology of SEI shares the appearance of classical EI but is restricted to the more superficial spinous and granular cell layers (Figure 7e) with possible intracorneal blister formation [14]. The distribution of granular degeneration is consistent with the expression site of keratin 2. 

#### 2.2.5. Incontinentia Pigmenti (IP) 

IP is a rare X-linked dominant genodermatosis caused by mutations in the inhibitor of the kappa B kinase gamma (IKBKG) (previously known as NEMO or nuclear factor kappa B essential modulator) gene. IP affects mostly female patients and is usually lethal in utero for males, but cases in boys are reported [15,16]. The phenotype in females is variable, manifestations being dependent on the effects of mosaicism resulting from X-chromosome lyonization.

**Figure 8:**
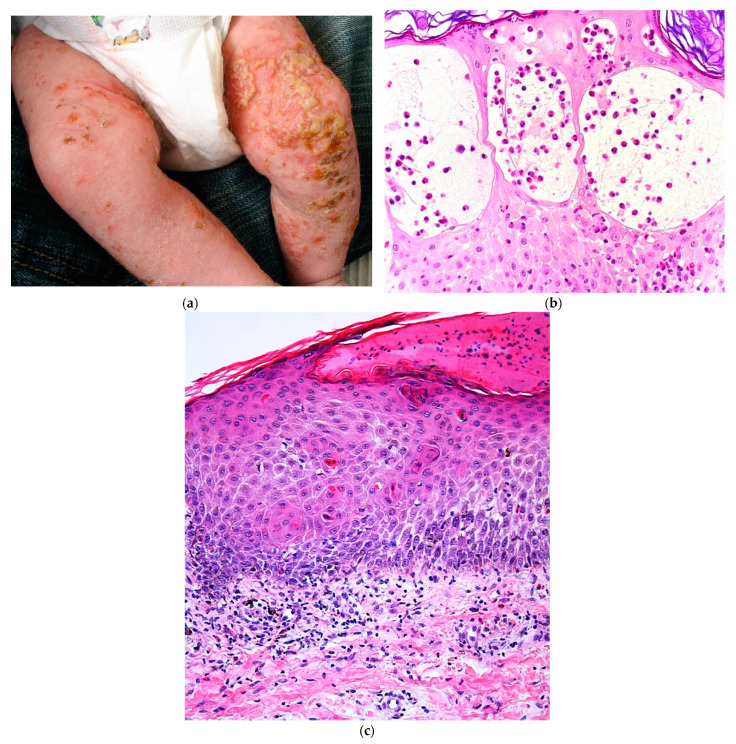
Incontinentia pigmenti. (**a**) Newborn with blistering Blaschko-linear eruption. (**b**) Hematein eosin ×40: spongiosis with eosinophils leading to vesicles. (**c**) Hematein eosin ×20: numerous apoptotic keratinocytes.

IP is a multisystem disorder of ectodermal origin and skin lesions may be associated with dental, ocular, and neurologic abnormalities. The skin is almost always involved in neonates. The lesions are classically vesiculobullous, following the Blaschko lines, and represent the first stage of cutaneous involvement. They are usually located on the limbs, trunk, and scalp (Figure 8a). Later stages are characterized by verrucous papules (stage 2), whorled hyperpigmentation (stage 3), and pallor and scarring (stage 4); different stages may overlap and only stages 1, 2, and 3 can be seen in infants. Skin histology is helpful for all 4 stages [17]. 

The differential diagnosis of IP lesions in newborns are herpes simplex infection, congenital varicella, and autoimmune blistering diseases, but the Blaschko-linear disposition of the lesion is helpful. Genodermatosis, including Conradi–Hunermann–Happle syndrome or Goltz syndrome, needs to be ruled out with histology. 

The skin biopsy is therefore always performed and of great help.

#### 2.2.6. Histology

At stage 1, eosinophilic spongiosis is observed, and its confluence leads to vesiculation and blisters (Figure 8b). Dyskeratotic/apoptotic keratinocytes (isolates or aggregates) are an important clue for diagnosis and can be assessed with serial cuts when necessary (Figure 8c). An inflammatory dermal infiltrate with eosinophils may be present. Free melanin can be observed in the dermis even at stage 1. In cases with an overlap between stages 1 and 2, hyperkeratosis, papillomatosis, and acanthosis can be present, as well as numerous apoptotic cells.

### 2.3. Cell Proliferation

#### 2.3.1. Mastocytosis

**Figure 9:**
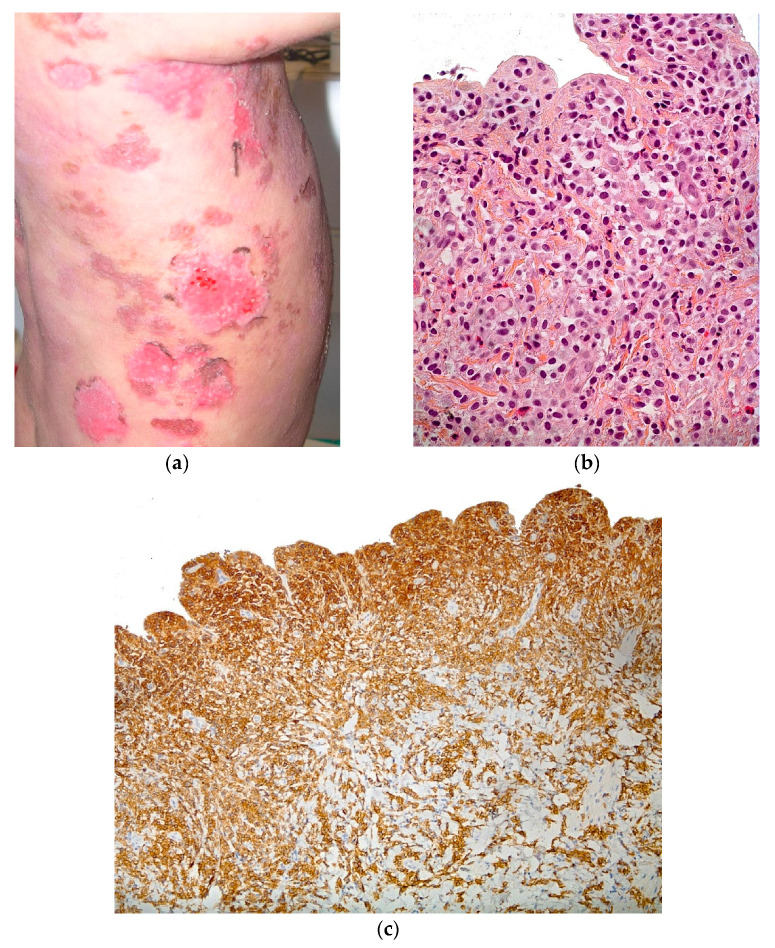
Mastocytosis. (**a**) Generalized blisters. (**b**) Hematein eosin ×40: floor of the blister, conservation of papillary relief, and dense mastocytes infiltrate. (**c**) Immunohistochemistry with anti-CD117 antibody ×10: positivity of the dermal infiltrate.

Mastocytosis is caused by a clonal mast cell proliferation associated with somatic activating mutations in *c-kit*. All forms of cutaneous mastocytosis may be associated with blistering in infancy, but the most frequent form associated with blisters is solitary mastocytoma. Clinical assessment will find a history of infiltrated red/purple lesions associated with the occurrence of blisters. Another very rare presentation is diffuse cutaneous mastocytosis (DCM) in a neonate having erythroderma and generalized blisters, and may be so serious that it mimics staphylococcal scalded skin syndrome (Figure 9a). The episodes of skin blisters are accompanied by pruritus and redness all over the body. The tendency to blister usually improves over 3–4 years [18,19,20]. 

#### 2.3.2. Histology

Skin biopsy shows a subepidermal blister surrounding a dense dermal mast cell infiltrate (Figure 9b). Immunohistochemistry is positive for the *c-kit* (anti-CD117) antibody (Figure 9c).

#### 2.3.3. Langerhans Cell Histiocytosis (LCH) 

**Figure 10:**
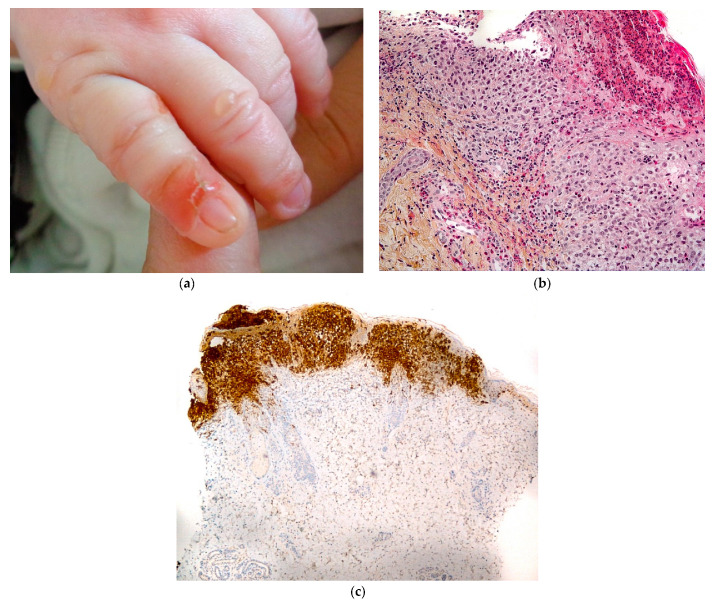
Langerhans cell histiocytosis. (**a**) Blister in a neonate. (**b**) Hematein eosin ×40: papillary infiltration with Langerhans cell, exocytosis resulting in blister and crusts, eosinophilic infiltrate in the dermis. (**c**) Immunohistochemistry with anti-CD1a antibody ×5: positivity of the dermal infiltrate.

LCH is a rare disease characterized by the aberrant clonal proliferation of Langerhans cells. Blistering presentation in cutaneous Langerhans cell histiocytosis, even if infrequent, may happen in the self-healing form (Figure 10a) [21,22,23,24]. 

#### 2.3.4. Histology

Langerhans cells (LC) have coffee-bean or kidney-shaped nuclei. The histiocytic infiltrate is epidermotropic and also situated in the papillary dermis, sometimes extending to the reticular dermis. The blister is situated at the dermo-epidermal junction. In the dermis, the infiltration is often associated with eosinophils and lymphocytes (Figure 10b). 

Detection of LC markers (CD1a and CD207 on formalin-fixed sample) is mandatory to confirm the diagnosis (Figure 10c). 

#### 2.3.5. Auto-Immune Blistering Diseases 

##### Bullous Pemphigoid (BP)

**Figure 11:**
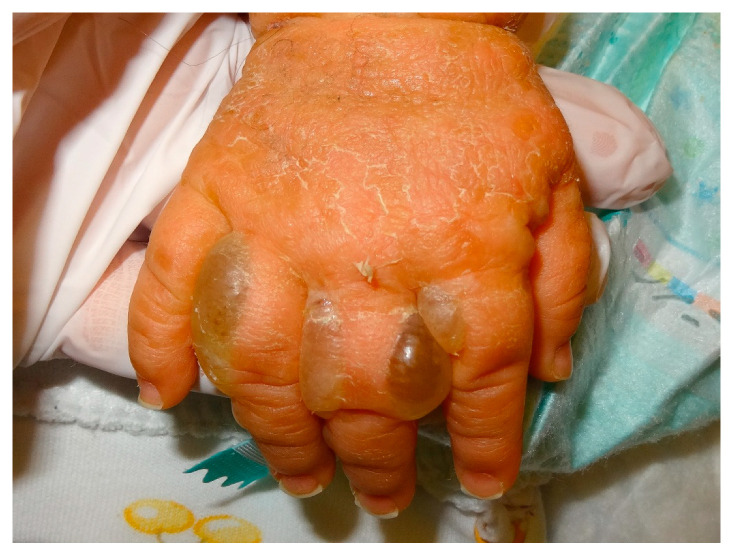
Bullous pemphigoid with typical appearance of bunch of grapes on the hand of an infant.

Bullous pemphigoid (BP), usually seen in elderly populations, is a rare condition in children. Like in adults, tense serous fluid-filled or hemorrhagic bullae occur on an inflammatory base or urticated plaques. The eruption is widespread and mucous membranes are not affected. Acral involvement (palms, soles, and head) is more common in infantile BP, sometimes with a bunch of grapes appearance (Figure 11) [25,26,27].

#### 2.3.6. Histology

Skin histology is not different from BP in adults: subepidermal blistering is observed associated with an inflammatory infiltrate made of eosinophils and often neutrophils. 

Histopathology shows eosinophilic spongiosis with clefting. DIF reveals linear deposition of IgG and/or C3 along the basement membrane. IgA deposition is also often seen.

#### 2.3.7. Other AIBDs

The other AIBDs are less frequent in this age group, since only about 50 cases of neonatal AIBDs were identified in a systematic review of the literature [28]. 

Pemphigus vulgaris, pemphigus foliaceus, bullous pemphigoid, pemphigoid gestationis, IgA-linear dermatosis, and epidermolysis bullosa acquisita have been reported in babies of affected mothers (antibodies are passively transferred from the mother to the neonate). Pemphigoid diseases are more likely to present after birth and were more predominant in males [25,26,27].

Skin histology does not differ from adult AIBDs.

#### 2.3.8. Drug Reactions 

Exposure to different medications can occur for the first time during infancy. Adverse drug reactions can have a blistering presentation, mostly in erythema multiforme (EM), Stevens–Johnson syndrome (SJS), and toxic epidermal necrolysis. 

In children, EM and SJS cases are often due to infections of herpes simplex virus (HSV) or *Mycoplasma*, while TEN is often associated with drugs [4]. 

As discussed above, TEN and SSSS can have a close clinical presentation and skin biopsy is useful to differentiate SSSS and TEN.

However, skin histology does not differ from adults for EM, SJS, and TEN.

## 3. Conclusions

The onset of blisters in a neonate or an infant is most often a diagnostic challenge for physicians, and the underlying cause has to be determined quickly between several etiologies.

The clinical context must be discussed with the clinician as well as the best technique for the biopsy.

Skin biopsy, often necessary, is particularly helpful for a rapid and precise diagnosis, leading to an appropriate management of the baby.

## Figures and Tables

**Table 1:** Most frequent blistering disorders in neonates and infants.

Infections	Staphylococcal scalded skin syndrome
	Impetigo
	Scabies
Genodermatosis	Hereditary epidermolysis bullosa
	Keratinopathic ichthyosis
	Incontinentia pigmenti
Cell proliferation	Mastocytosis
	Langerhans cell histiocytosis
Autoimmune blistering diseases	Bullous pemphigoid and others
Drug reactions	SJS, TEN, etc.

**Table 2:** Classical types of EB (adapted from Ref. [6]).

Level of Skin Cleavage	EB Type	Inheritance	Mutated Gene (s)	Targeted Protein (s)
**Intraepidermal**	EB simplex	Autosomal dominant	KRT5, KRT14	Keratin 5, keratin 14
			PLEC	Plectin
			KLHL24	Kelch-like member 24
		Autosomal recessive	KRT5, KRT14	Keratin 5, keratin 14
			DST	Bullous pemphigoid antigen 230 (BP230) (syn. BPAG1e, dystonin)
			EXPH5 (syn. SLAC2B)	Exophilin-5 (syn. synaptotagmin-like protein homolog lacking C2 domains b, Slac2-b)
			PLEC	Plectin
			CD151(syn. TSPAN24)	CD151 antigen (syn. tetraspanin 24)
**Junctional**	Junctional EB	Autosomal recessive	LAMA3, LAMB3, LAMC2	Laminin 332
			COL17A1	Type XVII collagen
			ITGA6, ITGB4	Integrin a6b4
			ITGA3	Integrin a3 subunit
**Dermal**	Dystrophic EB	Autosomal dominant	COL7A1	Type VII collagen
		Autosomal recessive	COL7A1	Type VII collagen
**Mixed**	Kindler EB	Autosomal recessive	FERMT1 (syn. KIND1)	Fermitin family homolog 1 (syn. kindlin-1)
